# Spatially Nonlinear Interdependence of Alpha-Oscillatory Neural Networks under Chan Meditation

**DOI:** 10.1155/2013/360371

**Published:** 2013-12-17

**Authors:** Pei-Chen Lo, Chih-Hao Chang

**Affiliations:** ^1^Department of Electrical Engineering, Institute of Electrical and Control Engineering, National Chiao Tung University, Hsinchu 30010, Taiwan; ^2^Institute of Electrical and Control Engineering, National Chiao Tung University, Hsinchu 30010, Taiwan

## Abstract

This paper reports the results of our investigation of the effects of Chan meditation on brain electrophysiological behaviors from the viewpoint of spatially nonlinear interdependence among regional neural networks. Particular emphasis is laid on the alpha-dominated EEG (electroencephalograph). Continuous-time wavelet transform was adopted to detect the epochs containing substantial alpha activities. Nonlinear interdependence quantified by similarity index *S*(**X**∣**Y**), the influence of *source* signal **Y** on *sink* signal **X**, was applied to the nonlinear dynamical model in phase space reconstructed from multichannel EEG. Experimental group involved ten experienced Chan-Meditation practitioners, while control group included ten healthy subjects within the same age range, yet, without any meditation experience. Nonlinear interdependence among various cortical regions was explored for five local neural-network regions, frontal, posterior, right-temporal, left-temporal, and central regions. In the experimental group, the inter-regional interaction was evaluated for the brain dynamics under three different stages, at rest (stage R, pre-meditation background recording), in Chan meditation (stage M), and the unique Chakra-focusing practice (stage C). Experimental group exhibits stronger interactions among various local neural networks at stages M and C compared with those at stage R. The intergroup comparison demonstrates that *Chan-meditation brain* possesses better cortical inter-regional interactions than the *resting brain* of control group.

## 1. Introduction

For several decades, scientific exploration has corroborated the effectiveness of meditation practice on health promotion. Particular evidence includes the improvement of cardiovascular functions, immunity, and hormone-level regulation. In addition, meditation makes positive changes in the brain and mind, including the positive emotional states, better stress manipulation, enhanced mindful attention, noticeable anxiety reduction, and depression relief, [1–7].

During the past decades, a number of meditation techniques have been developed and practiced all over the world. Although with somewhat different practicing scheme, almost all the practices are aimed to better manipulate the mind, brain function, and physical state of practitioners through mindfulness concentration and respiratory regulation. For many centuries, eastern religious and secular groups, such as the Buddhists, Taoist traditionalists, and the Indian Yogis, have been practicing meditation in order to achieve certain physical, mental, and spiritual realm. Meditation is a unique state of transcendental consciousness beyond the normal mind and mental process. Meditation may induce a series of integrated physiological changes. Among the diverse types of meditation, most practitioners are able to experience complete relaxation and the so-called tranquil awareness.

Although individuals in the East have been practicing various forms of meditation throughout history, scientific study of meditation did not begin until it became popular in the West. In recent years, meditation having been extended to complementary medical practices further motivated scientific studies with the focus of physiological alterations induced by the process [8, 9]. Increasing reports of meditation benefits further draw attention of researchers to the assessment of meditation in different indications. The research for physical and psychological correlation of meditation has been concentrated mostly on Yoga and transcendental meditation (TM) from India, Japanese Zen, and Tibetan Buddhism [10, 11]. Up to the present, little has yet been disclosed regarding the phenomena of Chan-Buddhist meditation (or simply “Chan meditation”). In the past decade, orthodox Chan meditation, as an unconventional therapy, has proved to be efficacious for many chronic diseases, infections, and even some malignant tumors. Consequently, more people began to practice orthodox Chan meditation in Taiwan. Accumulation of the effective evidences and health benefits of Chan meditation aroused our attention to the physiological investigation on the Chan-Buddhist disciples.

Since meditation process involves different states of mental activities and consciousness, EEG (electroencephalograph) thus became our major focus for exploring the human life system under Chan meditation. EEG applications in clinic and medical centers have become favorable since the 1970s because of its advantages of economy, safety, and convenience. Most of all, more scientific evidences of EEG variations have been disclosed in a number of different physiological, pathological, conscious and mental states in accordance with the various temporal, spectral, and spectral EEG characteristics. Although with the rapid progress in sophisticated medical imaging technologies, EEG still plays an important and irreplaceable role in long-term monitoring of brain functions exhibited as the lump variations of electrical activities. New findings have been continuously observed and reported [12–15]. As normally characterized by frequency, the EEG patterns are conveniently classified into five frequency ranges including delta (Δ, below 4 Hz), theta (*θ*, 4–8 Hz), alpha (*α*, 8–13 Hz), beta (*β*, 13–30 Hz), and gamma (*γ*, 30–70 Hz). Earlier paper [16], based on EEG spectral power and coherence estimates, reported the brain regions involved in meditative states as the selective associations of theta and alpha oscillating networks activity with states of internalized attention and positive emotional experience. According to our preliminary results, differentiation in frontal/occipital alpha activities plays a key role in comparing EEG between Chan-meditation practitioners (Appendix) and normal, healthy non-meditating subjects.

To explore the spatial interactions among brain local neural networks under alpha-rhythmic oscillation, methods developed in nonlinear dynamical theory become more versatile and favorable [17, 18]. The interactions among separate brain regions play a significant role in understanding the neurophysiological behavior of human brain. Accordingly, multivariate time series analysis based on nonlinear dynamical modeling becomes much appealing to investigate the important mechanism by which specialized cortical and subcortical regions integrate their activities into different functions and different spatial scales [19–23]. In recent studies, brain dynamics can be conceived as a large ensemble of coupled nonlinear dynamical subsystems. We have focused on investigating the nonlinear, chaotic characteristics of Chan-meditation EEG during the past decade, based on nonlinear deterministic modeling of brain dynamics [14, 24]. Significant nonlinear synchronization has been detected between the macroscopic scale of EEG channels. Thus, various types of synchronization based on the concepts of nonlinear dynamical systems theory have previously been proposed as a more powerful mechanism than narrow band frequency synchronization (e.g., coherence function) for achieving integrative neural processing. This type of “nonlinear coupling” allows studying nonlinear interdependence between multichannel recording sites and represents an alternative to the coherence function which addresses all of these limitations simultaneously [24–32]. As a consequence, this study aims to probe into the *α*-wave nonlinear interdependence behaviors among multichannel electroencephalograph (EEG) signals collected from the orthodox Chan-Buddhist practitioners (experimental group) and normal, healthy subjects (control group).

## 2. Material and Methods

### 2.1. Voluntary Subjects and Procedures

This study involved two groups of subjects, the experimental group including 10 volunteers with an average of 5.8-year Heart-Sealing Chan-meditation experience and the control group including 10 volunteers without any meditation experience. Seven men and three women were in either group. In the experimental group the average age was 28 years, while in the control group the average age was 23 years. Heart-Sealing Chan-meditation practitioners participated in one 90-minute group meditation session every week and practiced approximately 30-minute individual meditation on a daily basis. Heart-Sealing Chan meditation has been the only orthodox way of inheriting the lineage of Chan sect. The core essence of orthodox Chan-meditation practice is to transcend the physiological (fifth), mental (sixth), subconscious (seventh), and Alaya (eighth) states of consciousness and finally attain the realm of true self characterized by the pure golden light with eternal wisdom (Appendix). All the consciousness-transcending preprocesses inside can be completed for a well experienced orthodox Chan-meditation practitioner who has been able to spontaneously activate ten Chakras in our body [33]. These ten Chakras are important energy spots for altering states of consciousness by converting our physically and mentally dominant characteristics to a particular state of detachment. Accordingly, novice practitioners put lots of effort into the practice from Chakra focusing, Chakra perception, up to Chakra sealing. In the beginning stage of Chakra focusing, practitioners may practice the special *brain-drilling* technique to reduce all the wandering thoughts abiding in the brain. The *brain-drilling* technique involves, firstly, focusing alternately on frontal and posterior regions of the brain with mindfulness attention and, next, focusing alternately on left and right regions and perceiving the interconnections between two regions.

**Figure 1:**
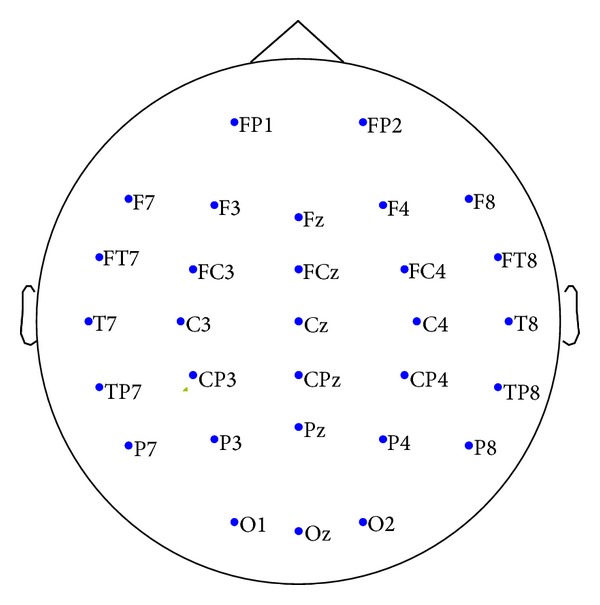
EEG electrode locations of the 30-channel recording montage.

In the experiment, we conducted overall 50-minute recording of EEG signals for both groups. The EEG signals were recorded by the 30-channel, common-reference (linked-mastoid MS1-MS2) electrode montage based on the international 10–20 system.  Figure 1 illustrates the EEG recording montage of the 30 electrode locations. The protocol designed for the experimental group involved three sessions: 5-minute premeditation relaxation (stage R), 40-minute Chan-meditation practice (stage M), and 5-minute Chakra focusing (stage C). In stage C, practitioners focused their mind and perception on a particular chakra named Chan Chakra (the third ventricle inside the brain, as illustrated in Appendix). No particular intervention was applied to the control group during the 50-minute EEG recording. The control subjects only sat in a relaxing position with eyes being closed, yet in the awake state.

### 2.2. Signal Acquisition and Preprocessing

The EEG signals were originally sampled at 1,000 Hz after being filtered by the analog, instrumentational band-pass filter with a passband of 0.5–50 Hz. The band-pass filter setting was selected to eliminate the 60 Hz interference by the power lines. A high sampling rate of 1,000 Hz was adopted to preserve the waveform quality of gamma rhythms (>25 Hz) often observed in Chan-meditation EEG that had been investigated in the other study of our research group. In this study, we downsampled the EEG with a sampling rate of 200 Hz since the major focus of this study is the alpha-dominated EEG epochs. The segments contaminated by such artifacts as eye blinking, eyeball movement, and muscle activities were prescreened in the preprocessing stage.

Wavelet decomposition provides an effective tool to extract the particular EEG rhythm of interest [34–36]. In addition, wavelet transform (WT) possesses such appealing properties as time-frequency localization and multirate filtering. Specific EEG rhythm may be extracted by dedicatedly designing the WT parameters. Wavelet transform can be implemented either in continuous configuration (CWT) or in discrete form (DWT). Due to the problem of extremely narrow-band EEG rhythmic pattern, CWT (continuous-time wavelet transform) was implemented in our study to reliably localize the correct spectral components of alpha rhythm.

In CWT, the signal to be analyzed is matched and convolved with the continuous wavelet basis function with the continuous time and frequency. The original signal is expressed as a weighted sum of the continuous basis wavelet function digitized by the sampling rate of the corresponding scale. The basis for wavelet transform is called the mother wavelet prototype. Wavelet functions are families of functions satisfying prescribed conditions, such as continuity, zero-mean amplitude, and finite or near finite duration. Some categories of wavelet functions may involve such properties like orthogonality and biorthogonality, regularity, and so forth [35–37].

Mother wavelet prototype needs to be appropriately selected according to the properties of the particular signal under investigation. Adeli et al. [38] successfully captured and localized the 3 Hz spike and wave complex in the epileptiform EEG by applying wavelet decomposition with Daubechies wavelets. Our previous study has corroborated the feasibility of adopting Daubechies 6 (DB6) wavelet as the mother wavelet in EEG rhythmic analysis [37].

The family of Daubechies wavelets is known for its orthogonal property and efficient implementation. The lower-order Daubechies wavelets are too coarse to properly represent EEG sharp transients. The higher-order ones with extra oscillations are beyond the requirements for analyzing the low-frequency EEG rhythms. Particularly, order 6 Daubechies wavelet becomes most appealing in our study because its waveform pattern appears to mimic the neuronal action potentials.

### 2.3. Nonlinear Interdependence Measure

The scheme for evaluating the nonlinear interdependence was based on the modified algorithm employed in computing the *similarity index S*(**X**∣**Y**) [24]. Major tasks involved in the algorithm are reconstruction of the *m*-dimensional phase-space trajectory and computation of the average cloud radius centered at a given state point.

#### 2.3.1. Reconstruction of *m*-Dimensional Trajectory

Consider the brain as a nonlinear dynamical system. The nonlinear interactions of the local neuronal networks can be assessed by the analysis of the collective dynamics underlying EEG time series simultaneously recorded from different brain regions. The first step is to reconstruct the multidimensional phase-space portrait of the system dynamics **X** and **Y,** respectively, from EEG time series *x*[*i*] and *y*[*i*]. According to the Takens embedding theory [39], a smooth map from the EEG time series {*x*[*i*] | *i* = 1,…, *N* + (*m* − 1)*τ*} to the phase-space trajectory **X** = {*X*_*i*_∣*X*_*i*_=(*x*[*i*],*x*[*i*+*τ*],…,*x*[*i*+(*m*−1)*τ*])}_*i*=1_^*N*^ preserves some important topological invariants of the original system. The reconstruction assumes a total number of *N* system-state points in the *m*-dimensional phase-space trajectory, utilizing a rational time delay *τ* (in sample point) [40, 41]. The dimension *m* indicates the number of degrees of freedom of the nonlinear system and, accordingly, reflects the *complexity* of the system dynamics.

#### 2.3.2. Computation of the Average Cloud Radius

**Figure 2:**
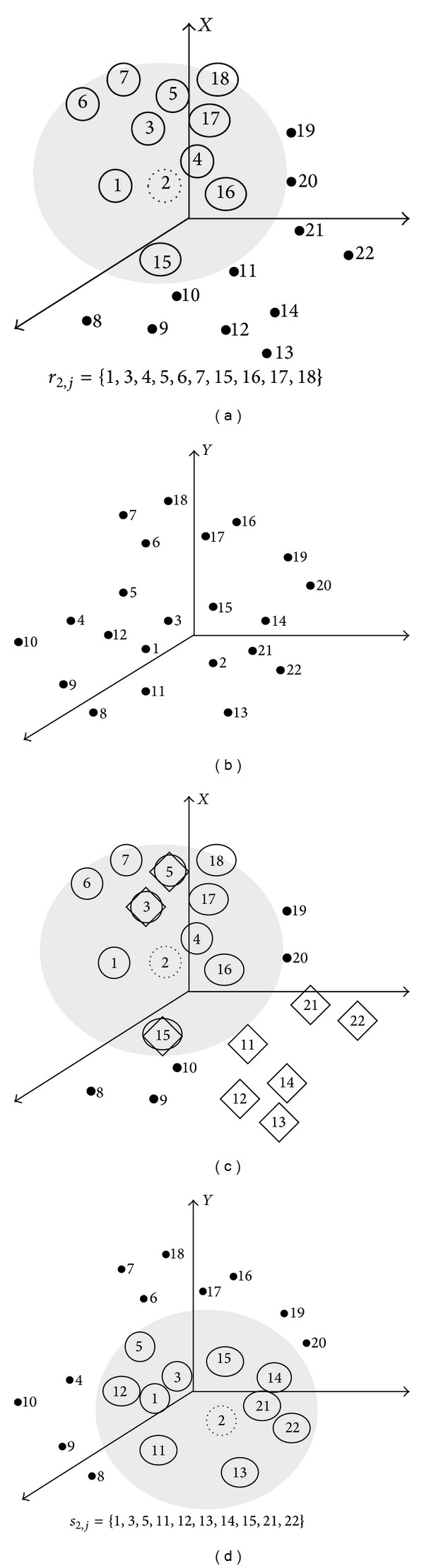
Illustration for (a) self neighbors *X*_*r*_2,*j*__ (◯), (b) state points in *Y*, and (c) mutual neighbors *X*_*s*_2,*j*__ (*♢*) where the indexes *s*_2,*j*_ are determined from the indexes of (d) *K*NN of *Y*_2_ (*K* = 10), assuming *m* = 3, *K* = 10, *i* = 2, and *N* = 22.

Consider a state point *X*_*i*_ on the *m*-dimensional phase trajectory. As illustrated in Figure 2, a *K*NN hypersphere, formed by the *K*'s nearest neighboring (*K*NN) points of **X**_*i*_, is a cloud composed of *Km*-dimensional neighboring points around *X*_*i*_. Let *r*_*i*,*j*_ and *s*_*i*,*j*_, *j* = 1, …, *K*, denote the time indices of the *K*NN points of *X*_*i*_ and *Y*_*i*_, respectively. Then, the set of state points in the *K*NN hypersphere centered at *X*_*i*_ is {*X*_*r*_*i*,*j*__ | *j* = 1,…, *K*}. The average square Euclidean distance from *X*_*i*_ to its *K*NN neighbors (or the average square radius of the cloud centered at *X*_*i*_) is defined as

(1) $$\begin{array}{c} R_{i}^{\left(K\right)}\left(X\right)=\frac{1}{K}\sum_{j=1}^{K}{||X_{i}-X_{r_{i,j}}||}^{2}, \end{array}$$

where ||·|| indicates the operator for calculating the Euclidean distance. Another point cloud around *X*_*i*_ is formed with respect to its *mutual* neighbors *X*_*s*_*i*,*j*__, which share the same temporal indexes of the *K*NN of *Y*_*i*_. In this sense, the **Y**-conditioned average square Euclidean distance is defined by replacing the true nearest neighbors of *X*_*i*_ by the *mutual* neighbors [37]:

(2) $$\begin{array}{c} R_{i}^{\left(K\right)}\left(X\;|\;Y\right)=\frac{1}{K}\sum_{j=1}^{K}{||X_{i}-X_{s_{i,j}}||}^{2}. \end{array}$$

In the extreme case of *K* = *N*, the average square radius of the trajectory centered at *X*_*i*_ is given by

(3) $$\begin{array}{c} R_{i}^{}\left(Χ\right)=\frac{1}{N-1}\sum_{j=1,j\neq i}^{N}{||X_{i}-X_{j}||}^{2}. \end{array}$$

Then, for two strongly synchronized systems, both self and mutual neighbors mostly coincide so that *R*_*i*_^(*K*)^(*X*) ≈ *R*_*i*_^(*K*)^(*X* | *Y*) ≪ *R*_*i*_(*X*); whereas for independent systems, mutual neighbors are more scattered that leads to *R*_*i*_^(*K*)^(*Χ*) ≪ *R*_*i*_^(*K*)^(*Χ* | *Y*) ≈ *R*_*i*_(*X*). Accordingly, the degree of interdependence of these two systems is reflected by the similarities (or dissimilarities) between these two cloud patterns formed by self and mutual neighbors. The strength of similarity between these two point clouds is termed as similarity index *S* [24, 37] and is defined as follows:

(4) $$\begin{array}{c} S^{\left(K\right)}\left(X\;|\;Y\right)=\frac{1}{N}\sum_{i=1}^{N}\frac{R_{i}^{\left(K\right)}\left(X\right)}{R_{i}^{\left(K\right)}\left(X\;|\;Y\right)}. \end{array}$$

*S* ^(*K*)^(*X* | *Y*) assesses the statistical dependence of the state-space structure of **X** on that of **Y** in the sense of testifying whether closeness in **X** implies closeness in **Y** and vice versa. Two identical systems with the same sets of self and mutual neighbors result in the maximum similarity index (*S* = 1), whereas the index is close to zero (*S* ≈ 0) for completely independent systems. The opposite interdependence (*S* ^(*K*)^(*Y* | *X*)) can be computed analogically. Notice that similarity indexes are in general asymmetric; that is, *S* ^(*K*)^(*Y* | *X*) ≠ *S* ^(*K*)^(*X* | *Y*). *S* ^(*K*)^(*X* | *Y*) evaluates the effect of system **Y** on system **X**. From the point of view of the system theory, signal **Y** is regarded as the *source* or the active role in the interaction, while signal **X** plays a passive role (a *sink*). On the other hand, *S* ^(*K*)^(*Y* | *X*) analysis considers **Y** as the *sink* that plays the passive role [24, 37].

The asymmetry of *S* is one of the main advantages over the other nonlinear measures such as the mutual information and the phase synchronizations. The fact that *S* is asymmetric allows us to study not only topographic patterns but also functional properties. By considering each EEG electrode either as a sink or as a source in the nonlinear-interdependence interaction, we may thereby further explore the brain functional topological profile and the direction of interaction among local neuronal networks [19]. For example, the condition of *S*(*Y* | *X*) > *S*(*X* | *Y*) indicates that **Y** depends more on **X** than vice versa. In other words, **X** has a greater influence on **Y** than vice versa. In such a case, **X** is said to be more *active* and **Y** is more *passive*. By considering each electrode either as a sink or as a source in the nonlinear dynamical interaction, we may thereby explore the spatial direction of the interaction and the dominance of local neuronal networks under Chan meditation [42].

In order to maximize the sensitivity to the underlying synchronization and gain the robustness against noise, we proposed a modified version of *S* measure with an adjustable range of *K*NN. Following our previous study of dimensional complexity index [27, 28], a reliable estimate of dimensional complexity of a system was obtained by averaging the complexity indexes over a moderate range of *K'*s. A small *K* causes superimposed noise, while a large *K* results in a measurement involving multimodal effects [27]. To determine a robust measure against noise, it follows that the final estimate of nonlinear interdependence is the average *S* ^(*K*)^(*X* | *Y*) over an appropriate range of *K*'s and is denoted by *S*(*X* | *Y*).

In the practical implementation, previous studies of dimensional complexity for meditation EEG have established a moderate choice of parameters. The time delay *τ* can be determined by the first zero-crossing of the corresponding autocorrelation function. Embedding dimension *m* can be determined by the convergent estimate of dimensionality. The window length *N* is selected to encompass the stabilization of dynamical behavior in the phase space in the sense of the convergent estimate of quantitative nonlinear dynamical property of reconstructed EEG trajectory, for example, correlation dimension. As a consequence, the implementing parameters were selected to be *τ* = 5 (sample points), *m* = 10, and window length *N* = 1, 000 sample points (5 seconds) that ensure convergent and reliable estimates [24, 27, 28]. The final estimate, *S*(*X* | *Y*), was obtained by averaging the *S* ^(*K*)^(*X* | *Y*) for *K* ranging from 20 to 35.

#### 2.3.3. Outline of the Scheme

**Figure 3:**
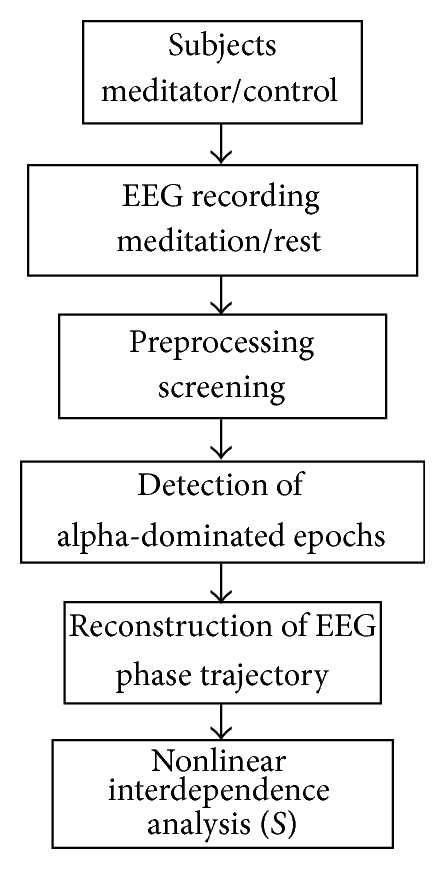
Scheme for evaluating nonlinear interdependence of multichannel EEG.

The entire scheme employed in this study is illustrated in Figure 3 that integrates different theories and methods to evaluate the nonlinear interdependence for multichannel EEG.

**Figure 4:**
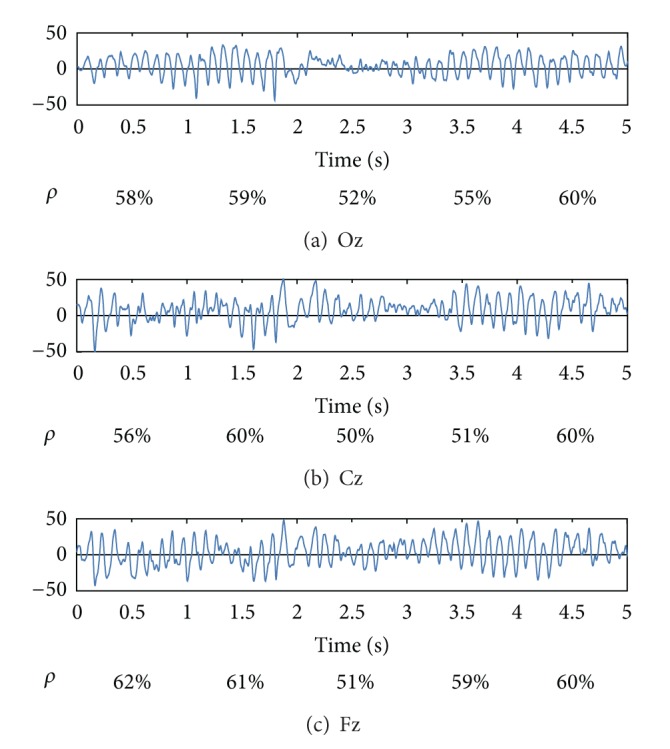
Percentage of alpha power to total power for each one-second epoch of the five-second EEG segments (amplitude in *μ*V) recorded from (a) Oz, (b) Cz, and (c) Fz.

To investigate the nonlinear-interdependent behaviors of alpha activities, CWT is employed to identify alpha-dominated epochs in the entire EEG record. An EEG segment is identified to be alpha dominant if the percentage of *α* power to the total power is at least 50% in more than 15 channels (one half of the total channels).  Figure 4 displays the results of interpreting the 5-second EEG recorded from channels Oz, Cz, and Fz. The alpha-power percentage (denoted as *ρ*) for each one-second epoch is listed beneath the EEG tracing. The 5-second EEG tracing is plotted with the amplitude ranging from −50 *μ*V to 50 *μ*V. Parameter *ρ* evaluated for different channels may reflect the focalized behavior of alpha activity.

To extend the capacity of assessing the neural-network interaction, the *source X*_*j*_ can be generalized as an integrated local network involving *L* *active* electrode sites so that *S*_*p*_(*X*_*i*_) becomes the average of *L*'s *S*(*X*_*i*_ | *X*_*j*_), assuming *X*_*j*_ ≠ *X*_*i*_:

(5) $$\begin{array}{c} S_{p}\left(X_{i}\right)=\frac{1}{L}\sum_{j}S\left(X_{i}\;|\;X_{j}\right). \end{array}$$

Equation (5) then evaluates the integrative effects of *L active* electrodes on *X*_*i*_. On the other hand, the influence of an *active* electrode *X*_*j*_ on the integrative neural network encompassed by *L* *passive* electrodes (*X*_*i*_) can be evaluated by

(6) $$\begin{array}{c} S_{a}\left(X_{j}\right)=\frac{1}{L}\sum_{i}S\left(X_{i}\;|\;X_{j}\right) \end{array}$$

assuming *X*_*i*_ ≠ *X*_*j*_. Both *S*_*p*_(*X*_*i*_) and *S*_*a*_(*X*_*j*_) are called *regional interdependence index* (*RII*).

In Chan-meditation practice, practitioners often focus on five regions alternately, frontal, posterior, left, right, and central regions, after activating the *Chan Chakra *inside the third ventricle. The purpose is to eliminate the stream of jumbled thoughts and produce a tranquil mind. To investigate the effect of such regional focusing, we accordingly divided 30 EEG recording sites into five regions:

 frontal (F): Fp1, Fp2, F7, F3, Fz, F4, and F8;
 posterior (P, Parietal + Occipital): O1, Oz, O2, P7, P3, Pz, P4, and P8;
 central (C): FCz, Cz, and CPz;
 left Temporal (LT): FC3, FT7, T7, C3, TP7, and CP3;
 right Temporal (RT): FC4, FT8, T8, C4, TP8, and CP4.

## 3. Results and Discussion

### 3.1. Interdependence Matrix of Chan-Meditation EEG

**Figure 5:**
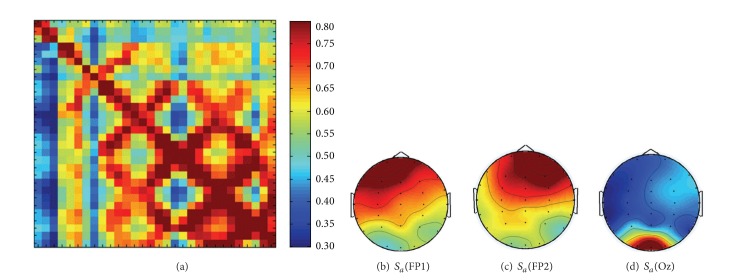
S.I. analysis for experienced practitioner during Chan meditation (Stage M) (a) 30 × 30** S** matrix, and brain topographical mappings (top view) of (b) *S*_*a*_(FP1), (c) *S*_*a*_(FP2), and (d) *S*_*a*_(Oz), indicating the average driving strength of the EEG sites FP1, FP2, and Oz.

Consider a given *source* signal **Y**. The influence of *source* signal **Y** on *sink* signal **X**, *S*(**X** | **Y**), can be expressed as a 30 × 30 interdependence matrix with the element *S*_*ij*_ = *S*(*X*_*i*_ | *Y*_*j*_) denoting the coupling strength of interaction of the source *Y*_*j*_ affecting the sink *X*_*i*_. The similarity index (S.I.) was calculated for 870 (30 × 29) electrode pairs. As displayed in Figure 5(a), the color image encoded the quantities in the 30 × 30 interdependence matrix **S**. The right-side color chart encodes the strength level of *S*_*ij*_, from blue to red indicating the range of *S* from the smallest to the largest value. EEG channels are in the order of (from top/left): O2, Oz, O1, P7, P3, Pz, P4, P8, TP8, CP4, CPz, CP3, TP7, T7, C3, Cz, C4, T8, FT8, FC4, FCz, FC3, FT7, F7, F3, Fz, F4, F8, Fp2, and Fp1. For example, the box at the lower-left corner characterizes the effect of O2 channel on Fp1 channel, as denoted by *S*(Fp1 | O2). Accordingly, the first row reveals the effect of *source* O2, Oz,…, and Fp1, respectively, on *sink* O2. On the other hand, the first column indicates how *source* O2 affects *sink* O2, Oz,…, and Fp1, respectively. The dark red along the diagonal line indicates the highest similarity index *S* = 1 when the *source* and *sink* signals are identical.

This figure exhibits some typical behavior in the *S* matrix; that is, stronger interdependence occurs in the pairs of nearby EEG channels. On the other hand, weaker interaction is measured as two channels are much apart. Moreover, box (*i*, *j*) does not equal its transposed partner box (*j*, *i*), indicating the asymmetry of *S* matrix. Figures 5(b)–5(d) display the top view of brain topographic mapping of *S*_*a*_(FP1), *S*_*a*_(FP2), and *S*_*a*_(Oz) extracted, respectively, from the 30th, 29th, and 2nd columns of Figure 5(a). The topographic mapping was plotted by the function *topoplot.m* provided by EEGLab. The mappings exhibit the efficacy of the given channel acting as the *source* role. The results in Figures 5(b) to 5(d) reveal the right-frontal dominance. The occipital channels are comparably less active with respect to the frontal neuronal networks. Such weaker influence of occipital and posterior regions on the other regions can be clearly observed from the blue color dominating in the left three columns of **S** matrix (Figure 5(a)), corresponding to the *source* at O2, Oz, and O1.

### 3.2. Inter-Region Interdependence Analysis—Experimental Group

Inter-regional nonlinear interdependence was analyzed for EEG recorded in three different sessions (stage R, M, and C). Due to the premeditation *brain-drilling* practice described in previous section, we particularly focused on the left-right temporal (LT-RT) and frontal-posterior (F-P) neural-network interactions. For example, *S*(F → P) is computed by averaging all *S*_*a*_(*X*_*j*_) in (6) for all *X*_*j*_ ∈ F and *X*_*i*_ ∈ P to assess the integrative *source* effect of all electrodes in frontal region driving the posterior region. On the other hand, *S*(P → F) is computed by averaging all *S*_*a*_(*X*_*j*_) in (6) for all *X*_*j*_ ∈ P and *X*_*i*_ ∈ F when all the electrodes in the posterior region play the *source* role to drive the frontal region.

Table 1 lists the group averages and standard deviations of *S*(F → P), *S*(P → F), *S*(LT → RT), and *S*(RT → LT) at three experimental stages (R, M, and C). Evidently, *S*(F → P) is consistently greater than *S*(P → F) for all three stages. The *P* values (0.0055, 0.0031, and 0.0302) of paired sample *t*-test are all smaller than 0.05, that demonstrates the statistical significance of frontal-alpha dominance at all three stages. On the other hand, results of the left-right temporal analysis of nonlinear interdependence reveal no distinctly dominant role of the laterally neural-network operation, that is, *S*(LT → RT) ≈ *S*(RT → LT). The higher *P* values for the *S*(LT → RT)-*S*(RT → LT) paired *t*-test indicate no statistically significant difference between two sets of results. We may further infer the balancing operations between the left-brain and right-brain hemispheres.

Our results demonstrate that interactions between left and right hemispheres are much more intensive than the interactions between frontal and posterior regions, with *P* = 0.0016, considering all the experimental subjects at all three stages.

**Figure 6:**
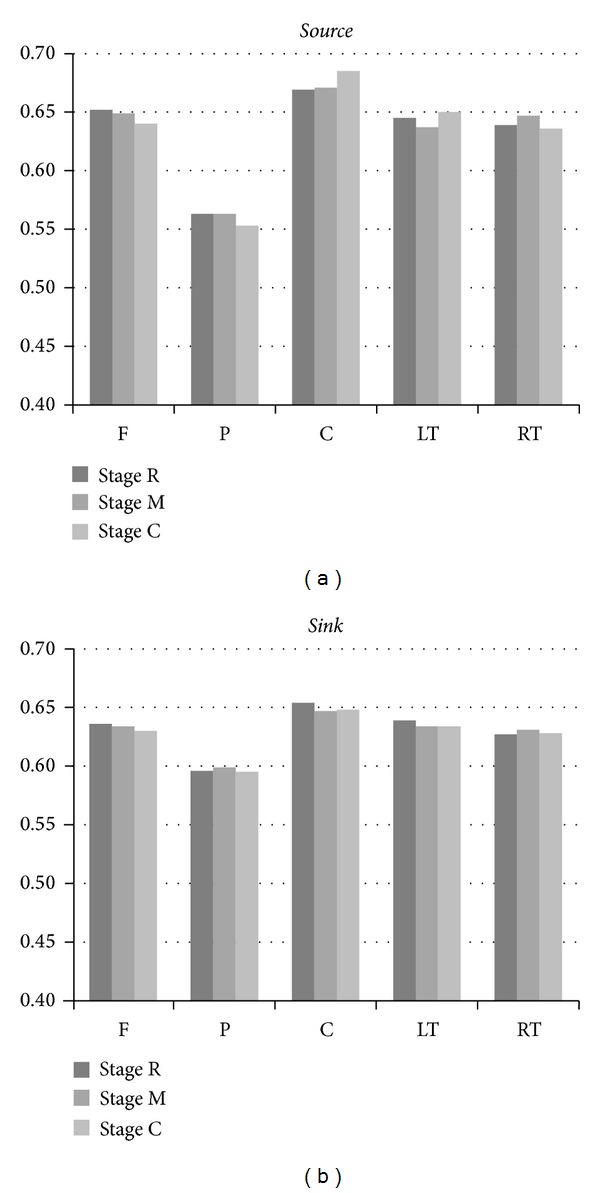
Average effectiveness of each region playing the role of a *source* (a) and *sink* (b). Three bars in each *RII* cluster correspond to three experimental stages.

Figure 6 provides an alternative viewpoint for exploring how a given region of interest ROI (F, P, C, LT or RT) influences or is influenced by the other regions. In Figure 6, left (right) group of five 3-bar clusters corresponds to the average effectiveness of each region playing the active (passive) role at three stages. For example, the leftmost bar indicates the average of *S*(F → P), *S*(F → LT),  *S*(F → RT),  and  *S*(F → C) for stage R, while the rightmost bar indicates the average of *S*(F → RT), *S*(P → RT), *S*(C → RT), and *S*(LT → RT) for stage C. Among all five regions, posterior region, as either the *source* or *sink*, apparently exhibits the weakest link to the other regions. In addition, the effectiveness of *active* role of posterior region is weaker than that of *passive* role. The results strongly suggest the inactive behaviors of parietal-occipital lobes since region P encompasses the EEG-electrode sites of parietal and occipital lobes. The parietal lobe is responsible for integrating sensory information from various parts of the body, with the particular functions of determining spatial sense and navigation. Functions of occipital lobe mainly include visual reception, visual-spatial processing, and color recognition. As described previously, the core essence of orthodox Chan-meditation practice is to transcend physiological, mental, and all states of consciousness to prove the existence of true being. The inactive posterior regions may provide the evidence of brain rewiring in preparation for such transcendence.

Region C encompasses three midline electrodes locating from precentral to postcentral cortex. Region C, as the *source*, apparently dominates over the other four regions regardless of the stages. On the other hand, region C as the *passive* role is affected mostly among the five regions. Region C constantly exhibits the largest *RII* at all stages.

We may draw a tentative hypothesis from the mechanism of Chan-meditation practice. Practitioners are required to keep Chan Chakra active at any moment, that results in the formation of an energy pathway between Chan Chakra and Qian-Ding acupoint on scalp (Figure 10(b)). Does such physiological reformation correlate to the significant effectiveness of region C? It leaves an open question for future investigation.

*RII* characterizing the regional interdependence behaves differently for each region when the experimental subjects switch their mental states from R (resting) to M (meditation) or from R to C (Chakra focusing). To investigate the effect of different experimental sessions, the *RII* percentage increase/decrease from stage R to M and from stage R to C were computed for each of the five regions (F, P, C, LT, and RT) acting as either the *active* or the *passive* role. In comparison of *RII* between stage M and stage R, the percentage larger than 1% was observed in the regions of LT_active_(−1.24%), RT_active_ (1.25%), and C_passive_ (−1.07%). On the other hand, the regions of significant change in *RII*, when comparing stage C with R, include F_active_ (−1.84%), P_active_ (−1.78%), and C_active_ (2.39%). On the basis of *RII* of stage R for each individual region, we summarize the changes of *RII* at stages M and C as follows.

1. In the *active*-role analysis, region LT becomes more deactivated at stage M, while region RT becomes more activated. When meditation subjects focused on Chan Chakra, the active driving strength of region C increases significantly (2.39%). On the other hand, suppression of the source activity occurs to both regions F and P (the regions anterior to and posterior to region C).
2. In the *passive*-role analysis, only region C becomes notably deactivated at stage M (free meditation). In general, differences are trivial in comparison of *RII*_passive_ between stages M and R.
3. Chan meditation deactivates the left brain hemisphere, whereas it inactivates the right brain hemisphere.

Except for region P, the *active*-role effectiveness of a given region is better than its *passive*-role effectiveness.

### 3.3. Inter-Region Interdependence Analysis—Control Group

In control group, the group average and standard deviation of *S*(F → P), *S*(P → F), *S*(LT → RT), and *S*(RT → LT) at stage R are, respectively, 0.55 ± 0.04, 0.51 ± 0.04, 0.59 ± 0.03, and 0.58 ± 0.04. The *P* values of student *t*-test for *S*(F → P)-*S*(P → F) and *S*(LT → RT)-*S*(RT → LT) pairs are 0.002 and 0.457. The results also reveal the frontal dominance and left-right lateral balance for the control group at rest. Yet, compared with the Chan-meditation practitioners, control group exhibits weaker strength of effectiveness no matter if the region plays an *active* or a *passive* role.

**Figure 7:**
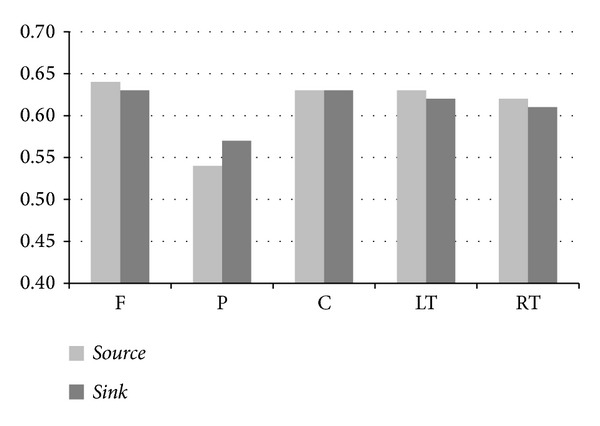
Average effectiveness of each region playing either the *active* or *passive* role.

To explore the average effectiveness of a given ROI, we averaged the *RII*s for the region in connection with the other four regions.  Figure 7 displays how a given ROI (F, P, C, LT, or RT) influences (*active*) or is influenced (*passive*) by the other regions. Similar to the results of experimental group, region P as either the *active* or *passive* role exhibits the weakest links to the other regions.

Comparing the efficacy of two counteractive roles played by the same region, we observe that the *source*-role effectiveness of a given ROI is higher than its *sink*-role effectiveness except region P.

### 3.4. Comparison between Experimental and Control Groups

**Figure 8:**
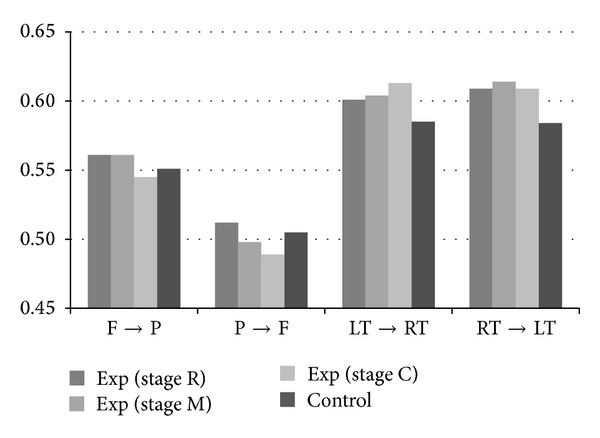
Group averages of *RIIs *(*S*(F → P), *S*(P → F), *S*(LT → RT), and *S*(RT → LT)) for experimental group at three stages and for control group at rest.

Figure 8 illustrates the group averages of *RIIs*, including *S*(F → P), *S*(P → F), *S*(LT → RT), and *S*(RT → LT), for the experimental group at three stages (R, M, and C) and for the control group at rest. Experimental group reveals much more intensive lateral (LT⟷RT) interactions than control group. The differences are statistically significant for experimental group at stage M (*P* = 0.0336) and at stage C (*P* = 0.0411). On the other hand, region P responsible for spatial sense and navigation becomes comparatively inactive for Chan-meditation practitioners at stage C.

**Figure 9:**
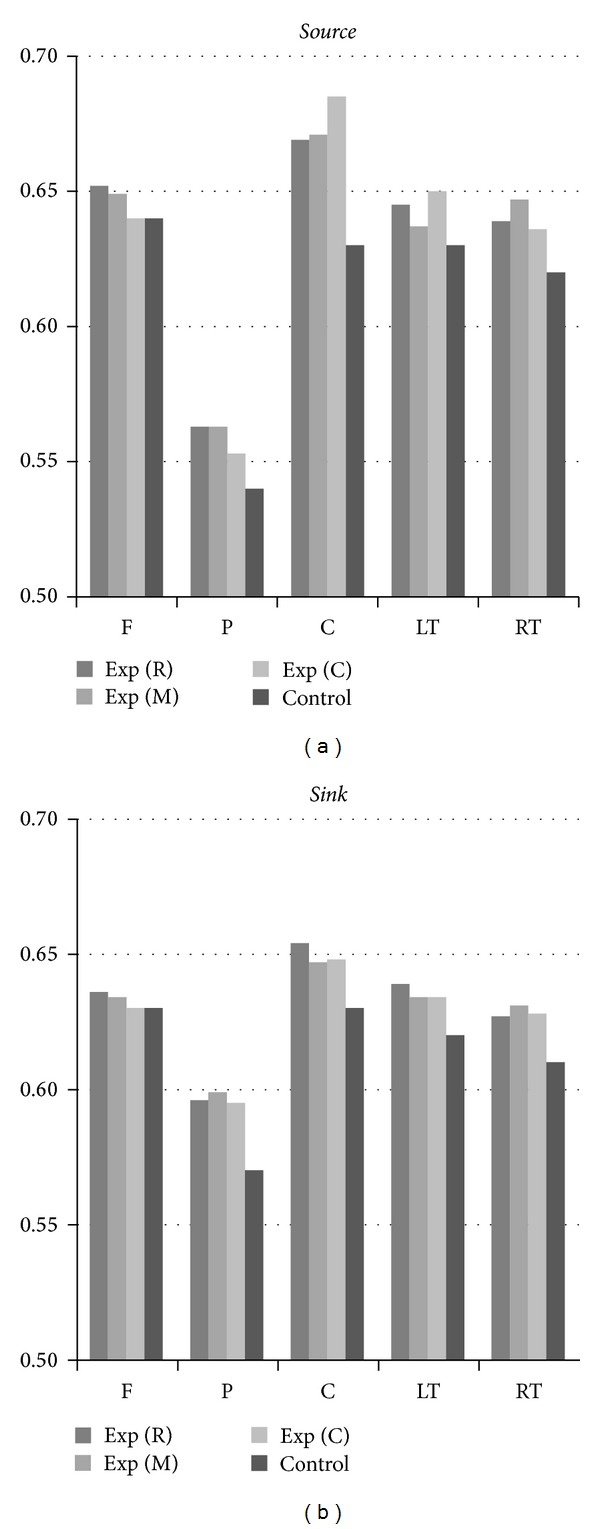
Comparison of average effectiveness of each region, as a *source* (a) or a *sink* (b), between experimental and control groups.

**Figure 10:**
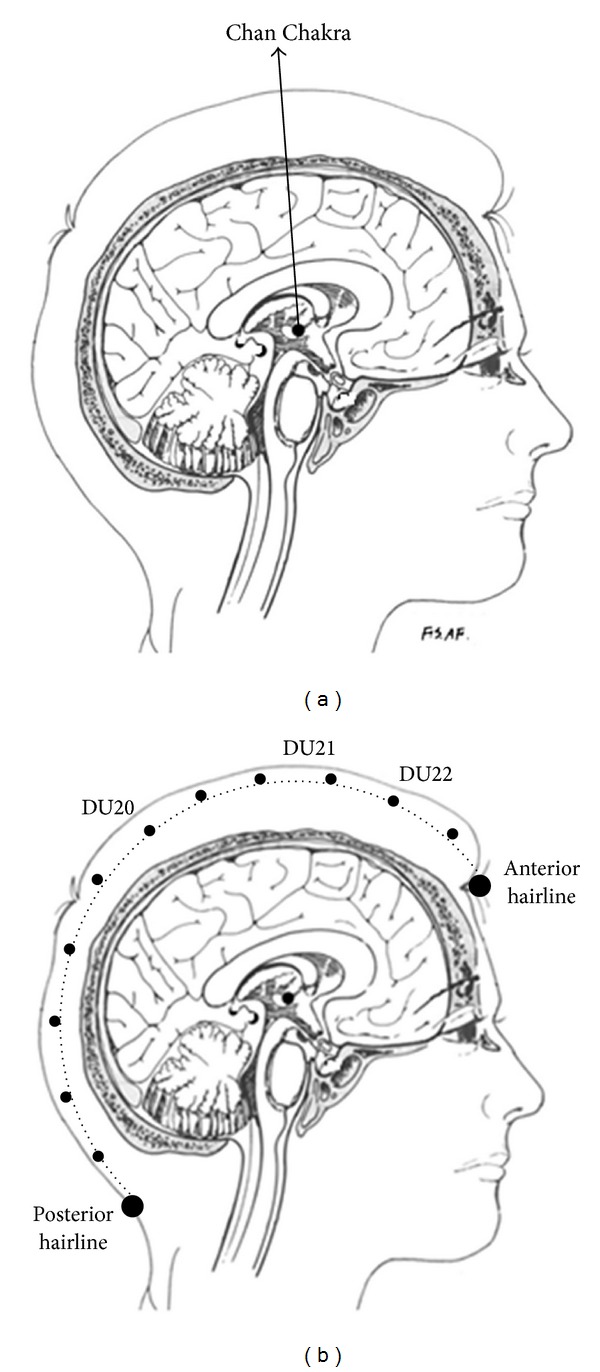
(a) Location of the Chan Chakra (inside the third ventricle). (b) Illustration of acupoints DU20 (Baihui), DU21 (Qianding), and DU22 (Xinhui) on DU meridian.

The assembling illustration in Figure 9 is used to compare the average effectiveness of each region between two groups. Experimental group, playing either a *source* role or a *sink* role, apparently exhibits higher average effectiveness in all five regions. The extraordinarily large *RIIs* for region C, particularly acting the *source* role, may be assumed to be correlated with the strengthening of neural networks of region C dominating over the other regions through the spiritual focusing on Chan Chakra. According to the postexperimental interview with Chan-meditation practitioners, such central (FCz-Cz-CPz) dominating behavior could be linked to the Chan-Chakra activation that further induces the perception of grand, solemn energy flow in and out through the cortical regions defined by acupoints DU20 (Baihui), DU21 (Qianding), and DU22 (Xinhui) in TCM (traditional Chinese Medicine). Figure 10(b) (Appendix) illustrates the locations of these three acupoints.

## 4. Conclusion

The time-transcending, nonmaterial sacred spiritual experiences of Chan-meditation practitioners bring our attention to the study of the unique interactions among regional neural networks in the brain. Scientific approach to the scope of Chan meditation provides insight into the mechanism in addition to the vague sketch of meditation sensation and its multiform benefits to human beings. This paper presents our preliminary results, based on nonlinear dynamical theory, of exploring the spatial interactions among brain local neural networks under alpha-rhythmic oscillation. Quantification of nonlinear interdependence based on similarity index reveals significant intergroup difference. Significant higher lateral interactions between left and right temporal regions were observed in Chan-meditation practitioners at the stages of Chan meditation and Chakra-focusing practice. In Chan practice, practitioners follow the doctrine that the mind can be enlightened only if it surrenders its leadership power to the “heart” (Bodhi, the true self with eternal wisdom). They accordingly can experience better balance and integration of the brain hemispheres through years of Chan-meditation practice.

Chan-Chakra spiritual focusing (at stage C) remarkably strengthens the central neural-network dominance over the other regions. On the other hand, suppression of the *source* activity in regions F and P at stage C appears to reveal the meditation state of transcending the realm of physical body and mind. The particular central (FCz-Cz-CPz) dominating phenomenon is reflected in long-term Chan practitioners as one of the metamorphosing processes that opens the energy pathway between Chan Chakra and the central-line scalp from acupoint DU20 to DU22 (Figure 10(b) in Appendix). In the case, practitioners experience tranquil brain and calm mind in every moment. Chan-meditation practice is to realize a Chan-style brain and Chan-style physical body, instead of merely sitting still for one hour to pursue temporary peace of mind and relief of body.

## Figures and Tables

**Table 1:** Group averages and standard deviations of *S*(F→P), *S*(P→F), *S*(LT→RT), and *S*(RT→LT) at three experimental stages (R, M, and C), including the *P* values of student *t*-test for *S*(F→P)-*S*(P→F) and *S*(LT→RT)-*S*(RT→LT) pairs.

Stage	*S*(F→P)	*S*(P→F)	*S*(LT→RT)	*S*(RT→LT)
R				
Group average	0.56	0.51	0.60	0.61
Group std	0.05	0.05	0.07	0.05
*P* value	0.0055		0.3476	

M				
Group average	0.56	0.50	0.60	0.61
Group std	0.03	0.06	0.06	0.04
*P* value	0.0031		0.1987	

C				
Group average	0.55	0.49	0.61	0.61
Group std	0.07	0.06	0.06	0.06
*P* value	0.0302		0.3953	

## Appendix

Chan meditation originating more than 2,500 years ago has been proved to benefit the health while on the way toward the ultimate Buddhahood state. Buddha Shakyamuni disclosed the eternal truth, the supreme wisdom, the noumenal energy, and the natural powers of the universe in Chan meditation under a linden tree. The orthodox Chan Buddhism was originated by such an exceptional affair that Buddha Shakyamuni transmitted this light of supreme wisdom to the Great Kashiyapa. The same path towards perfect enlightenment (Buddhahood) was promulgated in mainland China in 527 by Bodhidharma, the 28th patriarch. The current patriarch is Chan master Wu Jue Miao Tian, the 85th patriarch of the orthodox Chan-Buddhism Sect since the Great Kashiyapa. In orthodox Chan-Buddhist practice, very few disciples were able to catch the quintessence since it cannot be taught in any form of lectures. Written material and spoken words cannot promulgate the true wisdom of Chan, which can only be conveyed by the *Buddhist Heart-seal Imprint* from a true master.

In Chan meditation, practitioners aim to attain the *true self *(*Buddha nature*) with eternal wisdom (Bodhi) through body-mind-soul purification. Substantially speaking, such purification procedure involves the journal of transcending the physiological state (five sensory organs), the mental activities and normal consciousness, the subliminal (the manas) consciousness, and the Alaya state at which practitioners are able to perceive the sacred light emitted from *Buddha nature*. *Buddhist Heart-seal Imprint* from the Chan Patriarch is a must to assist in the purification and accomplishment. To prepare for attaining such realm, practitioners meditate with full-lotus, half-lotus, or leg-crossing posture and sit still to cultivate spiritual *Reiki* for penetrating into the ten important Chakras. In the course of Chan meditation, practitioners must switch their normal, chest breathing to the *Navel-Chakra* breathing (also called “fetal breathing”) that is the breathing scheme for entering into deep meditation. Among the ten Chakras, Chan Chakra locating inside the third ventricle is the Buddhist paradise implemented in our body.  Figure 10(a) illustrates the location of Chan Chakra.

The *cun*-measurand system is normally used to measure and locate the acupoints. To determine the locations of acupoints DU20, DU21, and DU22 on Governor Vessel meridian (DU meridian), we first measure the scalp-midline length between anterior hairline and posterior hairline, that is divided into 12 *cuns*. The locations are defined as follows:

DU20: 7 *cuns* above the posterior hairline and 5 *cuns* above the anterior hairline;
DU21: 3.5 *cuns* directly above the anterior hairline or 1.5 *cuns* anterior to DU20;
DU22: 2 *cuns* posterior to the anterior hairline or 3 *cuns* anterior to DU20.
